# Estimating chikungunya virus transmission parameters and vector control effectiveness highlights key factors to mitigate arboviral disease outbreaks

**DOI:** 10.1371/journal.pntd.0010244

**Published:** 2022-03-04

**Authors:** Frédéric Jourdain, Henriette de Valk, Harold Noël, Marie-Claire Paty, Grégory L’Ambert, Florian Franke, Damien Mouly, Jean-Claude Desenclos, Benjamin Roche

**Affiliations:** 1 Santé publique France (French National Public Health Agency), Saint-Maurice, France; 2 MIVEGEC, Université de Montpellier, IRD, CNRS, Montpellier, France; 3 Entente interdépartementale pour la démoustication du littoral méditerranéen (EID Méditerranée), Montpellier, France; 4 Santé publique France (French National Public Health Agency), regional office Provence-Alpes-Côte-d’Azur-Corse, Marseille, France; 5 Santé publique France (French National Public Health Agency), regional office Occitanie, Toulouse, France; Instituto de Salud Global de Barcelona, SPAIN

## Abstract

**Background:**

Viruses transmitted by *Aedes* mosquitoes have greatly expanded their geographic range in recent decades. They are considered emerging public health threats throughout the world, including Europe. Therefore, public health authorities must be prepared by quantifying the potential magnitude of virus transmission and the effectiveness of interventions.

**Methodology:**

We developed a mathematical model with a vector-host structure for chikungunya virus transmission and estimated model parameters from epidemiological data of the two main autochthonous chikungunya virus transmission events that occurred in Southern France, in Montpellier (2014) and in Le Cannet-des-Maures (2017). We then performed simulations of the model using these estimates to forecast the magnitude of the foci of transmission as a function of the response delay and the moment of virus introduction.

**Conclusions:**

The results of the different simulations underline the relative importance of each variable and can be useful to stakeholders when designing context-based intervention strategies. The findings emphasize the importance of, and advocate for early detection of imported cases and timely biological confirmation of autochthonous cases to ensure timely vector control measures, supporting the implementation and the maintenance of sustainable surveillance systems.

## Author summary

Dengue, chikungunya and Zika viruses have expanded their geographic range during recent decades and are now considered emerging threats in temperate areas. In particular, autochthonous transmissions of chikungunya virus (CHIKV) have regularly been observed in Europe since 2010. The increase in international travel and trade appear to be major factors, encouraging both a circulation of these viruses on a global scale and the dispersion of one of their main vectors, *Aedes albopictus*. This trend is likely to increase significantly in the future and improved preparedness and response strategies are essential to manage these emerging risks. In this respect of decision support, we developed a mathematical model for CHIKV transmission. We first estimated key model parameters of CHIKV transmission and vector control effectiveness, using data from the two main CHIKV transmission events which have already occurred in mainland France. The model was then used to forecast the magnitude of outbreaks as a function of the delay in implementing control measures, and from the moment of virus introduction during the mosquito vector season. This work will help provide stakeholders in public health with a greater understanding of the dynamics of CHIKV transmission, and with evidence for the implementation of sustainable surveillance systems.

## Introduction

Worldwide, arboviral diseases such as dengue, Zika and chikungunya constitute a major proportion of infectious diseases emergence [1]. In recent decades, their incidence has increased dramatically, and they have substantially extended their geographic range. While tropical countries bear the heaviest burden, some temperate areas are increasingly exposed to this threat due to the presence of *Aedes albopictus*, an efficient vector for their transmission. Various factors are likely to drive transmission in areas where *Ae*. *albopictus* is established and which have suitable environmental conditions [2].

In mainland France, various vector-borne transmission events have been observed over the past ten years [2,3]. Dengue fever events are the most frequent in the country [2,3], reflecting the global epidemiology of the disease [4]. However, transmission events of chikungunya virus (CHIKV) in France results in outbreaks with the greatest number of cases. This can be partially explained by the fact that *Ae*. *Albopictus* is considered as an efficient vector of CHIKV, especially East-Central-South African (ECSA) genotypes [5], whereas the species is not considered currently as a primary vector of dengue [6]. Moreover, a series of adaptive mutations of CHIKV are selected by *Ae*. *albopictus* for even better transmission, as highlighted during the major CHIKV outbreak in the Indian Ocean in 2005–2006 [7,8]. The main mutation—an alanine to valine substitution at position 226 of the E1 glycoprotein—was also present in the viral genotypes at the origin of the two main episodes of CHIKV transmission that occurred in mainland France in 2014 and 2017 [9,10]. For each of the two events, an imported case (primary case) was identified and in both situations, the imported case was returning from Cameroon [3].

This increase in CHIKV fitness in *Ae*. *albopictus*, combined with the strong capacity of the latter to invade new areas, including areas with temperate climates, explains the potential for major epidemics of CHIKV to occur throughout Europe. Accordingly, health authorities, especially in France, have proactively strengthen strategies for preparedness and response to arboviral risks [2]. To prevent local transmission a better understanding of the effectiveness of current surveillance and control strategies is needed, together with a better knowledge of the factors that influence it.

The potential for CHIKV transmission in temperate areas as well as the effectiveness of vector control remain poorly understood. The acquisition of such knowledge is essential to inform evidence-based decision-making regarding surveillance and control strategies. In this perspective, we developed a compartmental epidemiological model with a vector-host structure to estimate key model parameters of both CHIKV transmission and current vector control interventions, based on observations made during previous CHIKV transmission events. We then assessed the effectiveness of these strategies according to different virus transmission scenarios during the vector activity season.

## Methods

### Model

**Fig 1:**
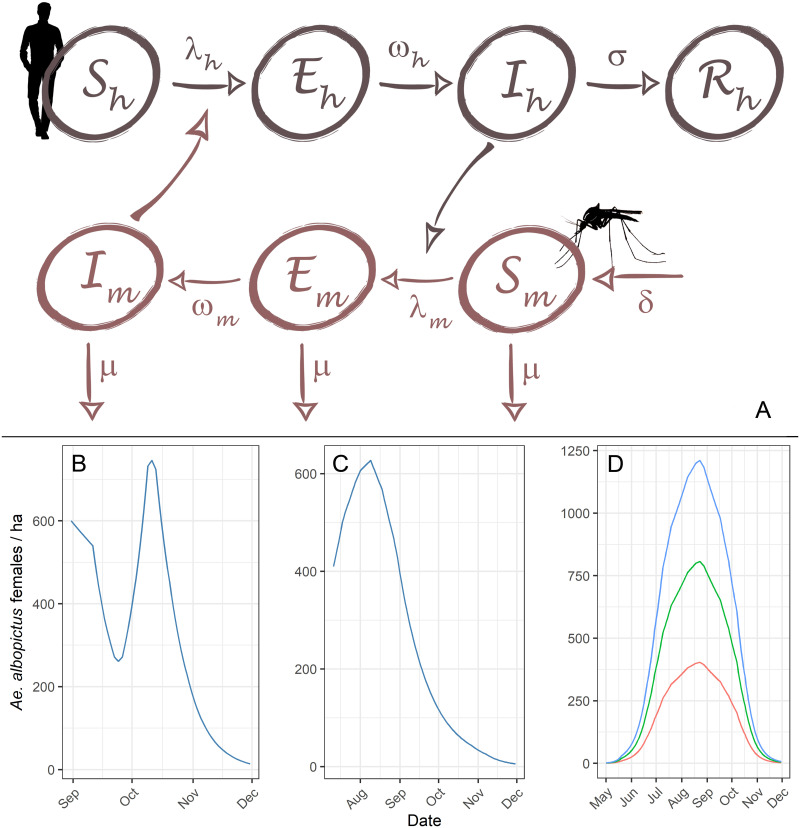
Structure of the model and vector population dynamics. (A) Structure of the SEI-SEIR vector-host model. The infection force of the hosts (*λ*_*h*_) and the infection force of the vector population (*λ*_*m*_) are respectively defined by the expressions $ab\frac{I_{m}}{N_{h}}$ and $ac\frac{I_{h}}{N_{h}}$. Lower panel: (B) vector population dynamics based on data from Montpellier (C) and from Le Cannet-des-Maures, and (D) standard vector population dynamics modelled throughout the whole period of vector activity for three different mosquito population densities.

We developed a mathematical model based on a Susceptible-Exposed-Infectious-Recovered host and Susceptible-Exposed-Infectious vector framework (Fig 1A). Vector demography was incorporated into the model in order to integrate the seasonal activity of *Ae*. *albopictus*. To this extent, the birth rate of mosquitoes was modulated to fit with the dynamics observed by entomological surveillance in Montpellier (Fig 1B), in Le Cannet-des-Maures (Fig 1C) and with the dynamics of an artificial standard mosquito population (Fig 1D).

The rate of recruitment (birth and immigration) and the mortality rate of human population were ignored in the model because of the short time scale of each transmission event. Neither was disease-induced mortality considered given the limited size of events. Humans start in a susceptible state (S_h_), and are then infected (E_h_) through bites of infectious mosquitoes (I_m_) at rate *a*, with the probability *b*. They become infectious (I_h_) at a rate ω_h_, finally recover (R_h_) at a rate *σ*, and then become immune. Similarly, susceptible mosquitoes (S_m_) biting infectious humans (I_h_) at rate *a*, can become infected (E_m_) with the probability *c* and then infectious (I_m_) at the rate *ω*_*m*_, the inverse of the extrinsic incubation period. The choice was made not to consider the presence of asymptomatic forms in order to have a model that was as simple as possible, while still meeting the objectives of decision support. This choice is justified by the significant effort of epidemiological investigation implemented in the area of virus circulation and was also reinforced by an analysis of the impact of the presence of asymptomatic forms on the estimates made (S1 Text).

We first used a deterministic model which is expressed by a set of ordinary differential equations. For the human compartments:

$$\frac{dS_{h}}{dt}=-ab\frac{S_{h}I_{m}}{N_{h}}$$ (1)

$$\frac{dE_{h}}{dt}=ab\frac{S_{h}I_{m}}{N_{h}}-\omega_{h}E_{h}$$ (2)

$$\frac{dI_{h}}{dt}=\omega_{h}E_{h}-\sigma I_{h}$$ (3)

$$\frac{dR_{h}}{dt}=\sigma I_{h}$$ (4)

For the vector compartments:

$$\frac{dS_{m}}{dt}=\delta N_{m}-ac\frac{S_{m}I_{h}}{N_{h}}-\mu S_{m}$$ (5)

$$\frac{dE_{m}}{dt}=ac\frac{S_{m}I_{h}}{N_{h}}-\left(\;\mu +\omega_{m}\right)E_{m}$$ (6)

$$\frac{dI_{m}}{dt}=\omega_{m}E_{m}-\mu I_{m}$$ (7)

The following expression was used to compute the basic reproduction number, $R_{0}$ [11]:

$$R_{0}=a2\frac{N_{m}}{N_{h}}\;\frac{bc}{\sigma \mu }\;\frac{\omega_{m}}{\omega_{m}+\mu }$$ (8)

Parameter values were taken from the literature (Table 1) with preference given to a selection of data adapted to *Ae*. *albopictus* and to the viral genotypes involved in the two transmission events [9,10].

**Table 1:** Description of parameters used in the chikungunya virus transmission model.

Parameter	Definition	value	Source
1/μ	Mosquito lifespan	10.5 days	[12]
a	Biting rate of mosquitoes	0.22	[13]
b	Human susceptibility to infection	To be estimated	-
c	Mosquito susceptibility to infection	0.67	[14,15]
1/ω_m_	Extrinsic incubation period	8 days	[16]
1/ω_h_	Intrinsic incubation period	3 days	[17]
1/σ	Recovery rate	6 days	[18,19]

To account for the stochasticity of both mosquito and human infections, we used, as a second step, a continuous-time stochastic version of the model, whose transitions and rates were specified by analogy with the deterministic equations (S1 Table). Numerical simulations were performed using Gillespie simulation algorithm.

Vector control strategy is mainly based on the use of adulticides with Ultra-Low Volume spraying of pyrethroids within a 250 m radius area around the residences of the cases, covering the entire area of viral circulation. Vector control was simulated by reducing the entire vector population of the area according to the effectiveness of the control measure (*Eff*, i.e. the proportion of the adult vector population reduced thanks to control measures) at the precise moment mosquito adulticide was sprayed. Such dynamics in vector population reduction thus reflect the immediate effect and the absence of persistence of insecticides used for urban vector control. Afterwards, the vector population returns after a few days to the level that would have been observed in the absence of control [20,21]. This dynamic covers not only the newly emerged mosquito adults, but also the recolonization by imagos present in the adjacent patches. It can be considered conservative, as it limits the effectiveness of vector control on disease incidence [21].

The model aimed to estimate two parameters: the efficacy of control measures, *Eff* and human host susceptibility to infection, *b* (i.e. the probability that a human host gets infected after being bitten by an infectious vector). To do this, the model was fitted using a Markov chain Monte Carlo (MCMC) method with the Metropolis-Hastings algorithm. The particle MCMC Metropolis-Hastings algorithm was used to evaluate the likelihood of stochastic models [22]. MCMC was run for five different chains with different initial values for both parameters in order to avoid converging towards local minima. Model fitting and simulations were performed with R [23] and, specifically the fitR package [24].

### Data

Epidemiological data were collected by the French national public health agency, during two autochthonous transmission events of CHIKV (S2 Table). These events occurred in 2014 in Montpellier [9] and in 2017 in Le-Cannet-des-Maures, both on the Mediterranean coast [10]. The two events led to an equivalent number of cases (12 and 11 cases in Montpellier and Le Cannet-des-Maures, respectively). However, the transmission event started rather late in the vector activity season in Montpellier (August 30) while it started in mid-season in Le Cannet-des-Maures (July 10) [25]. In both events, the primary (imported) case was identified and the CHIKV strain belonged to the ECSA genotype with the A226V mutation of the E1 protein. Following epidemiological investigations, the estimated total affected area of transmission was 7.1 and 8.4 ha, in Montpellier and Le-Cannet-des-Maures, respectively. Population density was estimated at 70 and 45 hab/ha, respectively. Active door-to-door investigations were conducted in both areas of virus circulation to identify symptomatic cases who had not consulted a physician [9,10]. Vector population dynamics in Le-Cannet-des-Maures were derived from the results of routine entomological surveillance conducted in 2017 in Nice (located 80 km from Le-Cannet-des-Maures) by the local public mosquito control agency (EID-Méditerranée) which used a network of 50 ovitraps. For Montpellier, we used surveillance data from a study conducted in the area in 2014 to describe mosquito population dynamics [26]. From previous studies dedicated to mosquito population density estimation [27] and nuisance perception during entomologic field investigations, we assumed a maximum mosquito population of 600 females/ha in Le Cannet-des-Maures and 730 females/ha in Montpellier. A standard vector population dynamic (for both events) was also derived from data collected in Nice by the public mosquito control agency between 2008 and 2017 in order to simulate the dynamics of vector populations usually observed in the South of France. For these standard population dynamics, we considered three different mosquito population densities with a maximum population size of 400, 800 and 1 200 females/ha in order to describe the different possible urban environments. Mosquito control measures were implemented at different time points during both local transmission events (S2 Table). Initial conditions for our model simulation were established on the basis of these data and are reported in S3 Table.

### Definition of scenarios

We tested the impact of *different scenarios* on the magnitude of outbreaks, using the estimated parameters. First, we considered the context of the outbreaks that occurred in both areas. To do this, we initially forecasted what the size of the outbreaks in Montpellier and Le-Cannet-des-Maures would have been in the *absence of any vector control action*. We then compared the hypothetical sizes of each outbreak according to *different timings of vector control action* (i.e. scenarios with earlier or later interventions with respect to the actual timing of interventions). More specifically, we simulated response delays in vector control implementation between 0 and 90 days after primary case introduction while keeping the number of treatments and the spacing between each treatment similar to what actually occurred during the transmission events in 2014 and 2017. Finally, for each of the two outbreaks, *different vector control sequences* were tested by varying the number and frequency (i.e., the sequence) of control measures.

Second, with a view to generalizing the model predictions to assess the impact of response delay and time of virus introduction during a standard vector activity period, we based our model simulations on a standard mosquito population dynamic (Fig 1D). Different simulations were run for three mosquito densities corresponding to low (maximum of 400 females/ha), medium (800 females/ha) and high density (1200 females/ha). In this context, reduced effectiveness of vector control was also considered since the value of the effectiveness of the vector control measures is not absolute. More specifically, effectiveness can be reduced because of various constraints and difficulties including limited access to the areas to be treated, absence of residents, physical barriers created by buildings, public opposition to insecticides, and unfavourable weather conditions. As part of this modelling framework, we also considered the impact of the sequences of vector control measures. In particular, we simulated different time points of primary case introduction throughout the activity season of *Ae*. *albopictus* in the South of France, from May 1 to November 30. For each outbreak, we simulated (i) different response delays between 20 and 90 days, (ii) 1 to 6 treatments, and (iii) intervals between treatments of 5, 7 and 10 days.

## Results

Infectious disease transmission is mainly a stochastic process, especially in the early stage of an epidemic. Hence, particular emphasis was given thereafter to the results of the stochastic model.

### Inference of parameters

The joint posterior distribution of parameters *Eff* and *b* was obtained from 5 chains of 5 000 MCMC iterations, with a burn-in period of 750 iterations for the deterministic version of the model. The 5 chains converged to similar posterior distributions for the two parameters and both geographical settings. The vector control efficacy was estimated at 97% (95% CI: 0.91–1) and 83% (95% CI: 0.78–0.89) in Montpellier and Le-Cannet-des-Maures respectively. The probability of host infection reached similar mean values for the two events: 0.37 and 0.33 in Montpellier and Le-Cannet-des-Maures, respectively (Table 2 and S1 Fig).

**Table 2:** Estimates of the efficacy of vector control measures (Eff), the probability of host infection (b) and the corresponding basic reproduction rate ($R_{0}$) for both chikungunya events.

Transmission event	*Eff*			Host infection probability (b)			$R_{0}$		
	Mean	5^th^ perc.	95^th^ perc.	Mean	5^th^ perc.	95^th^ perc.	Mean	5^th^ perc.	95^th^ perc.
Montpellier	0.97	0.91	> 0.99	0.34	0.33	0.35	1.86	1.83	1.88
Le-Cannet-des-Maures	0.83	0.78	0.89	0.29	0.28	0.31	1.78	1.72	1.84

**Fig 2:**
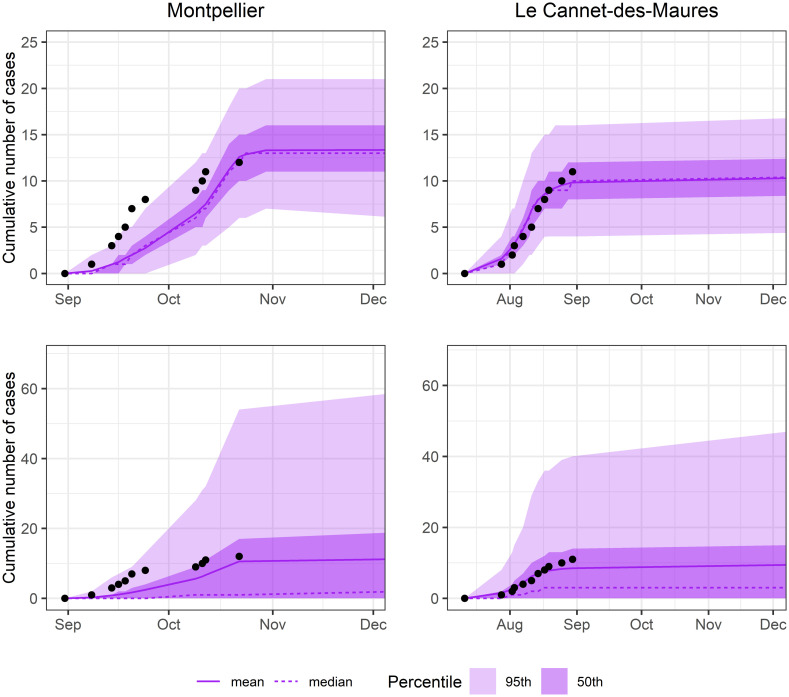
Simulation of the outbreak in Montpellier and Le Cannet-des-Maures using the parameter model estimates. The upper row shows the fit of the deterministic models while lower row shows the fit of the stochastic models.

The deterministic model provides a good fit for the data, especially for the event in Le Cannet-des-Maures. However, we were not able to fit the stochastic version of the model with particle filtering [22], probably due to the limited number of cases. Nevertheless, the simulations of the stochastic model using the parameters estimated by the deterministic approach provided a good fit of the data for both events (Fig 2). Based on these results, $R_{0}$ was estimated at 1.86 (95% CI: 1.83–1.88) in Montpellier and 1.78 (95% CI: 1.72–1.84) in Le-Cannet-des-Maures.

### Estimates of the size of the outbreaks according to different scenarios

#### Absence of vector control

Considering the previous estimates for both foci, we quantified the potential epidemiological impact of these two outbreaks in the absence of vector control measures by simulating 500 replicates. Accordingly, in Montpellier, the chikungunya outbreak would have reached 39 cases (0–178 CI 95%) without vector control according to the stochastic model. In Le-Cannet-des-Maures, the stochastic model forecasted a cumulative incidence of 114 cases (0–333 CI 95%) in the absence of vector control (Table 3 and S2 Fig). The simulations of these two different events illustrate the influence of the starting date of the outbreak on its potential size. Forecasts exhibit a large variation in the size of the outbreak due to the random nature of the stochastic equations and the high rate of extinction, as illustrated by the median values. The deterministic simulations display a higher mean number of cases with less variation in the results (Table 3 and S3 Fig).

**Table 3:** Number of cases estimated by the model for different scenarios of vector control.

	Scenario	Deterministic model				Stochastic model			
		Mean	Median	Percentile		Mean	Median	Percentile	
				5^th^	95^th^			5^th^	95^th^
**Montpellier**	t_1_ = 51, t_2_ = 56, t_3_ = 62 **(Base MPL, 3 VCM)**	12.8	13	6	20	11.3	2	0	59
	With no mosquito control measures **(0 VCM)**	47.8	48	35	62	39	2	0	178
	t_1_ = 51 **(1 VCM)**	16.1	16	9	24	13.3	2	0	79
	t_1_ = 51, t_2_ = 56 **(2 VCM)**	13.2	13	7	21	13.0	3	0	65
	t_1_ = 51, t_2_ = 62 **(2 VCM)**	13.3	13	7	21	12.3	1	0	63
	t_1_ = 51, t_2_ = 58, t_3_ = 65 **(3 VCM, 7 day frequency)**	12.8	13	6	20	11.2	3	0	57
	t_1_ = 51, t_2_ = 61, t_3_ = 71 **(3 VCM, 10 day frequency)**	13.3	13	7	21	11.2	1	0	59
	t_1_ = 46, t_2_ = 51, t_3_ = 57 **(5 days earlier than Base MPL)**	9.5	9	4	16	9.3	3	0	46
	t_1_ = 41, t_2_ = 46, t_3_ = 52 **(10 days earlier than Base MPL)**	7.2	7	2	13	6.5	1	0	33
	t_1_ = 56, t_2_ = 61, t_3_ = 67 **(5 days later than Base MPL)**	17.1	17	10	26	14.8	2	0	74
	t_1_ = 61, t_2_ = 66, t_3_ = 72 **(10 days later than Base MPL)**	21.8	22	13	31	18.7	3	0	98
	t_1_ = 71, t_2_ = 76, t_3_ = 82 **(20 days later than Base MPL)**	31.5	31	21	43	26.6	3	0	135
**Le Cannet-des-Maures**	t_1_ = 32, t_2_ = 39, t_3_ = 43, t_4_ = 50 **(Base LCM, 4VCM)**	10.2	10	4	17	10.8	2	0	65
	With no mosquito control measures **(0 VCM)**	197.0	197	170	225	114.2	45	0	333
	t_1_ = 32 **(1 VCM)**	76.0	76	59	94	51.0	4	0	228
	t_1_ = 32, t_2_ = 39 **(2 VCM)**	31.8	32	21	43	30.7	5	0	146
	t_1_ = 32, t_2_ = 43 **(2 VCM)**	30.2	30	20	41	25.2	2	0	139
	t_1_ = 32, t_2_ = 50 **(2 VCM)**	32.3	32	22	44	25.3	1	0	131
	t_1_ = 32, t_2_ = 39, t_3_ = 43 **(3 VCM)**	14.3	14	7	22	15.6	2	0	97
	t_1_ = 32, t_2_ = 39, t_3_ = 50 **(3 VCM)**	15.4	15	8	23	14.8	3	0	84
	t_1_ = 32, t_2_ = 43, t_3_ = 50 **(3 VCM)**	16.1	16	9	24	15.3	3	0	84
	t_1_ = 32, t_2_ = 39, t_3_ = 46, t_4_ = 53 **(4 VCM, 7 day frequency)**	10.7	11	5	19	10.7	3	0	55
	t_1_ = 32, t_2_ = 42, t_3_ = 52, t_4_ = 62 **(4 VCM, 10 day frequency)**	11.6	11	5	19	10.8	2	0	55
	t_1_ = 27, t_2_ = 34, t_3_ = 38, t_4_ = 45 **(5 days earlier than Base LCM)**	7.1	7	2	13	9.9	2	0	62
	t_1_ = 22, t_2_ = 29, t_3_ = 33, t_4_ = 40 **(10 days earlier than Base LCM)**	5.2	5	1	10	6.8	1	0	63
	t_1_ = 37, t_2_ = 44, t_3_ = 48, t_4_ = 55 **(5 days later than Base LCM)**	14.4	14	8	22	14.5	2	0	77
	t_1_ = 42, t_2_ = 49, t_3_ = 53, t_4_ = 60 **(10 days later than Base LCM)**	20.6	20	12	30	20.9	5	0	95
	t_1_ = 52, t_2_ = 59, t_3_ = 63, t_4_ = 70 **(20 days later than Base LCM)**	40.1	40	28	53	32.0	7	0	143

VCM: Vector control measure(s). For each scenario, VCM are performed at the different ti, expressed in number of days after primary case introduction. ‘Base MPL’ is the actual sequence of vector control measures implemented in Montpellier in 2014, whereas ‘Base LCM’ refers to the actual sequence of vector control measures implemented in Le Cannet-des-Maures in 2017. The primary case was introduced at t = 0 for both events.

#### Impact of response delay on outbreak size

**Fig 3:**
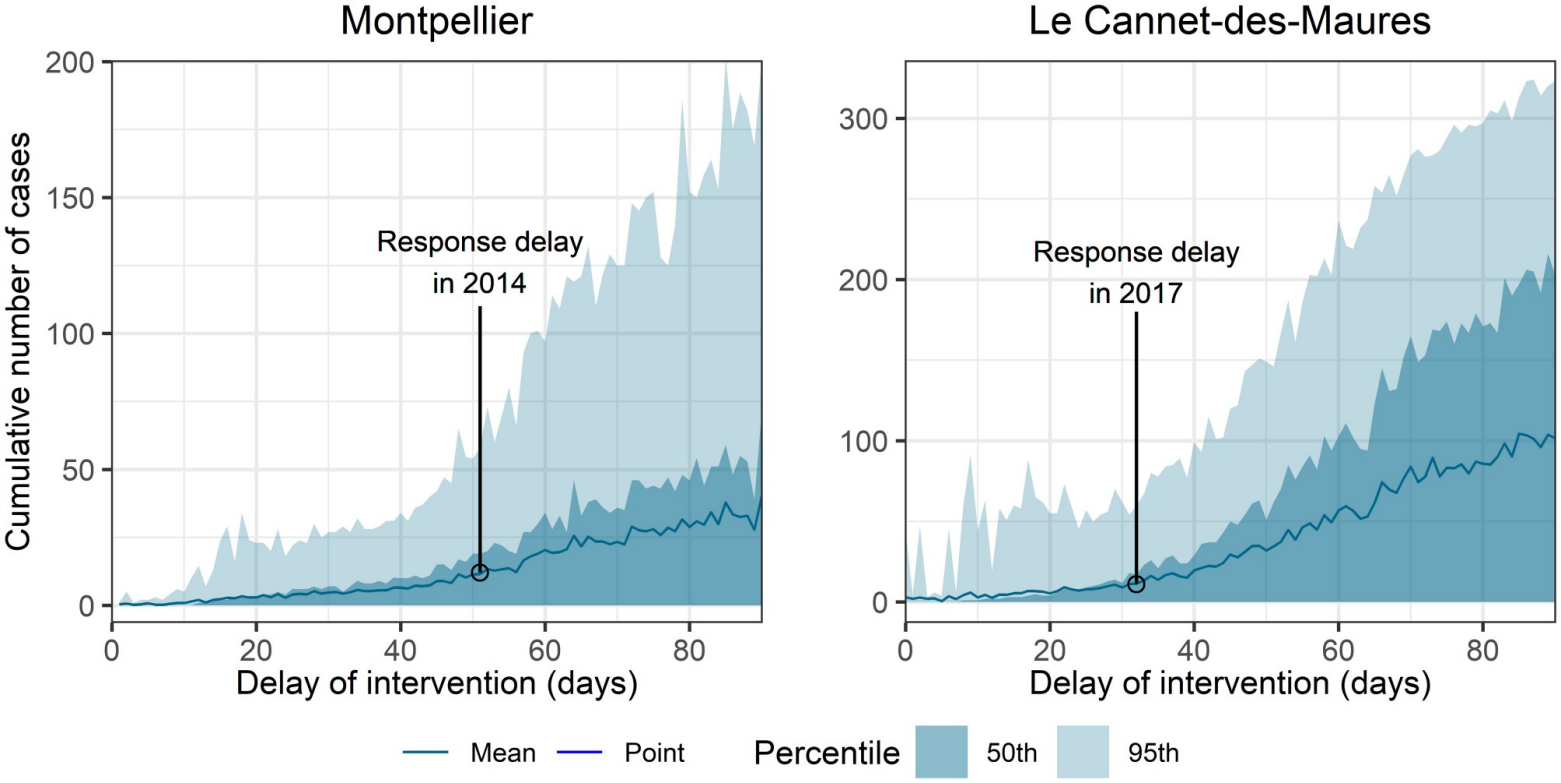
Stochastic simulations of the cumulative number of autochthonous cases according to the delay between the introduction of the primary case and control measure intervention. In Montpellier (left-hand column), vector control was first implemented 51 days after primary case introduction and 12 outbreak cases were reported, as marked by the circle in the figure. In Le Cannet-des-Maures (right-hand column), vector control was first implemented 32 days after primary case introduction and 11 outbreak cases were reported, as marked by the circle in the figure.

The sizes of the outbreaks were simulated for different delays of vector control implementation. Response times between 0 and 90 days after primary case introduction—as described in the scenarios introduced in the Methods section—were considered for both events (Fig 3). For instance, the implementation of measures 10 days earlier than when the interventions were actually performed in 2014 and 2017, would have resulted in 30–40% fewer cases for both events on average; a contrario, if the measures implemented had occurred 10 days later than the original interventions, there would have been on average 65% more cases in Montpellier and 100% more in Le Cannet-des-Maures (Fig 3 and Table 3). The comparison of the two events allowed us to assess both the impact of the response delay after primary case introduction and the impact of the date of primary case introduction on the size of the resulting outbreak. The delay in implementing control measures after the introduction of the primary case was longer in Montpellier (51 days later) than in Le Cannet-des-Maures (32 days). Both events reached a similar final size (12 and 11 cases in Montpellier and Le Cannet-des-Maures, respectively). However, the transmission event started at the end of the vector activity season in Montpellier (August 30) whereas it started in mid-season in Le Cannet-des-Maures (July 10) [25].

#### Impact of variations in vector control sequences

Different sequences of vector control were derived from the original sequences implemented in Montpellier in 2014 and in Le Cannet-des-Maures in 2017. Results of the different simulations based on these sequences are shown in Table 3. They illustrate the epidemiological benefit of successive treatments. Unlike the time interval between treatments, the number of treatments has a strong impact. The importance of the latter parameter is related to the occurrence of the event during the period with the highest risk as illustrated by the simulated scenarios at Le-Cannet-des-Maures, while the impact of the number of treatments is not as large for scenarios simulated for Montpellier as the event there took place at the end of the vector activity period.

### Estimates of the size of an outbreak for a standard vector population dynamic

**Fig 4:**
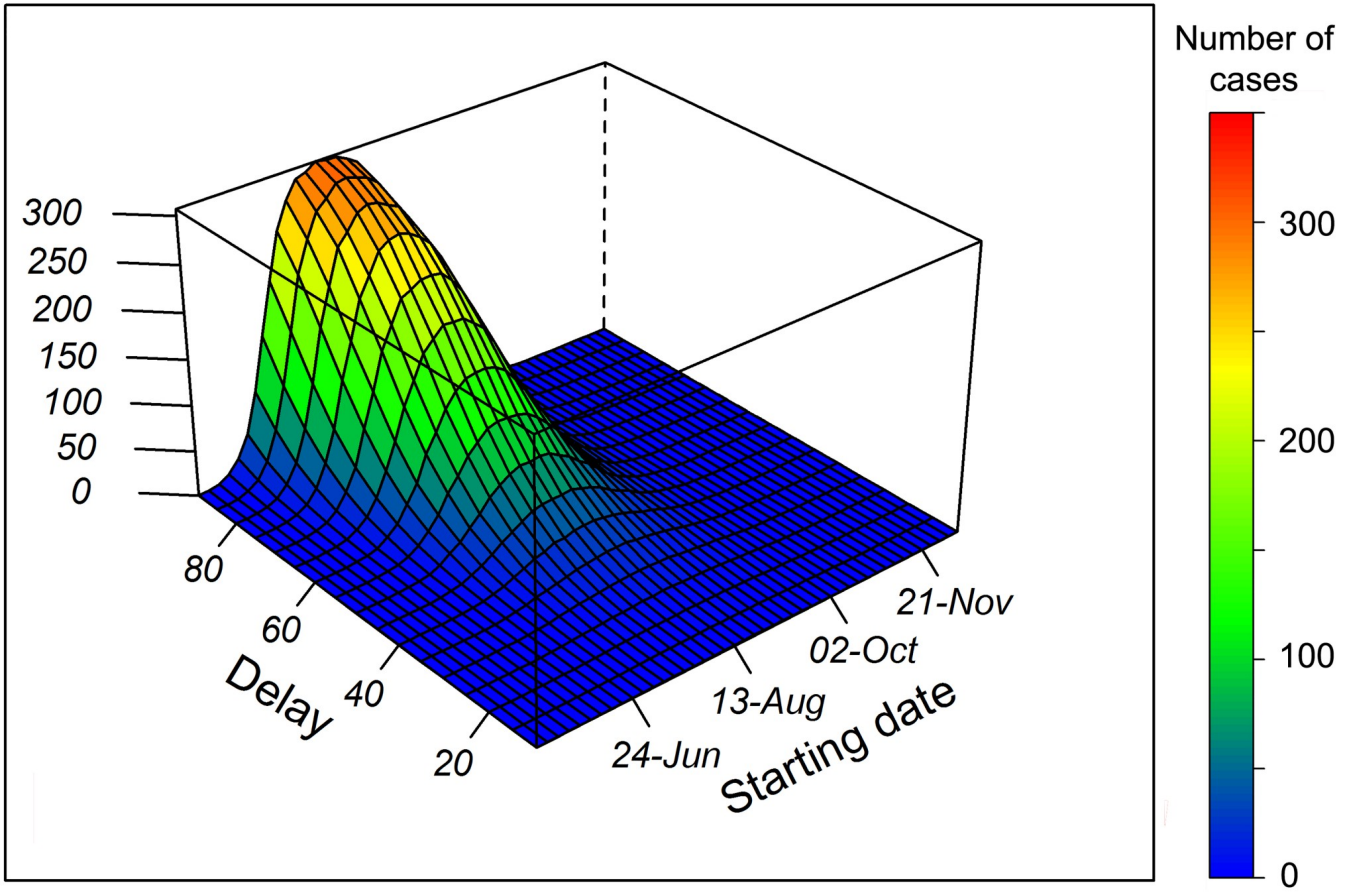
Number of autochthonous cases as a function of the date of primary case introduction and delay of intervention. Simulations are derived for a medium (800 females/ha) vector density of a standard mosquito population dynamic. For each setting, a sequence of 10 vector control treatments spaced 7 days apart is implemented.

Using the results outlined above for the specific two real-world events, we estimated the size of a hypothetical outbreak for a standard vector population dynamic (Fig 1D) in an attempt to generalize the previous results. We first studied the size of hypothetical transmission events as a function of the date of primary case introduction and of the response delay. We simulated three different vector densities and two values of vector control effectiveness. Our results suggest little influence of a reduction in vector control efficacy on the size of transmission events, provided that reduction was offset by an increase in the number of treatments. A contrario, vector density had a significant positive impact on outbreak size (S4 and S5 Figs). These simulations highlight the importance of rapid implementation of control measures, in particular during the peak season of vector activity, and are shown for a medium vector density with a vector efficacy of 90%, on the basis of the results of the estimates (Fig 4).

**Fig 5:**
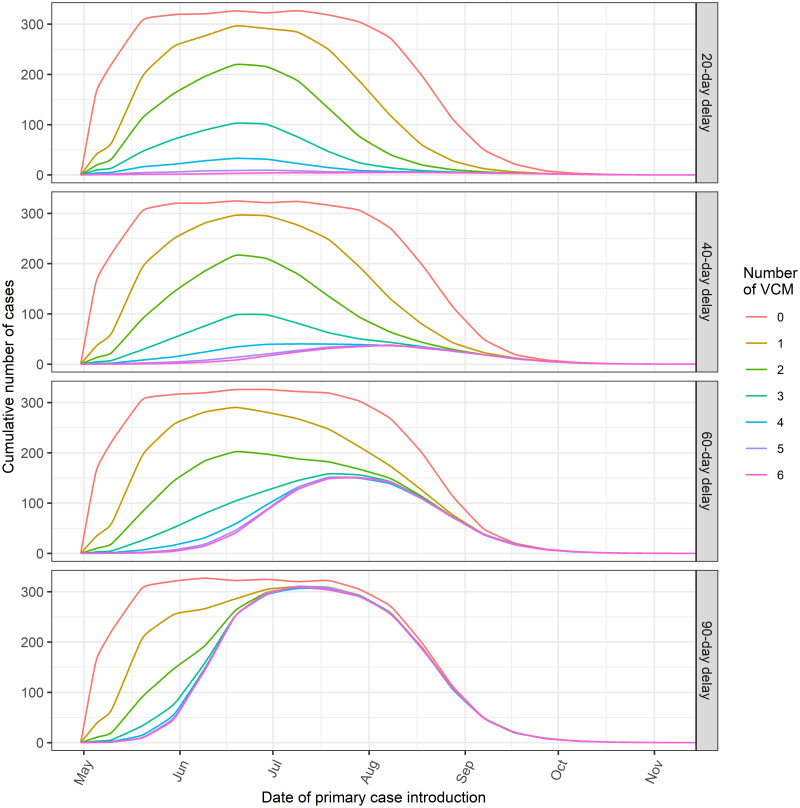
Number of autochthonous cases as a function of the date of virus introduction and different number of vector control measures. Simulations are performed for a standard mosquito population dynamic for four different delays in vector control implementation. VCM: vector control measure(s). The values on the y-axis correspond to the average number of cumulative cases expected during an entire event of transmission (until the end of the vector activity season), according to the date of introduction of the virus (x-axis).

Further simulations of vector control sequences (Fig 5) suggest that a single measure would have a limited impact. The optimal number of treatments in terms of vector population reduction is between 3 and 5. Our model indicates that the control of any hypothetical event of autochthonous transmission requires at least 3 treatments, or 4 or 5 if it occurs before the peak of seasonal vector activity. The impact of several successive treatments is, however, counterbalanced by the delay in response and, to a lesser extent, by the late occurrence of the virus introduction during the season. We varied the spacing between treatments to 5, 7 and 10 days, which corresponds to what is regularly implemented. A 5 days spacing is a reasonable frequency in terms of feasibility, but can sometimes be increased due to logistical or meteorological constraints. Such variation of the spacing between vector control treatments had little impact on the size of the outbreak (S6 Fig).

Simulations of virus transmission under standard conditions lead to qualitatively similar conclusions and support the above results based on real-world situations. Overall, this approach allows to generalize the conclusions for different vector densities and for virus introductions throughout the vector activity period.

## Discussion

In this work, we estimated transmission parameters and vector control effectiveness based on two chikungunya transmission events that occurred in mainland France using a compartmental epidemiological model. Estimated values were similar for both events with vector control effectiveness between 85 and 97%. While this result may seem quite high, it is consistent with the impact on vector population of the vector control measures actually implemented during the outbreak in Montpellier. Following the first insecticide treatment, the vector population declined drastically in the treated area, by 97% [9].

A very wide range of values is proposed in the literature for human susceptibility to CHIKV infections [28]. Our results are very close to those found by some authors [11,29–31]. Basic reproduction numbers were estimated at 1.86 in Montpellier (2014) and 1.78 and in Le-Cannet-des-Maures (2017). Our estimates of $R_{0}$ are the first for CHIKV transmission in mainland France. They add important results to the very fragmentary current level of knowledge about the transmission of arboviruses in temperate climates. A recent review of the basic reproduction number for dengue, Zika and chikungunya across global climate zones, proposed an average $R_{0}$ estimate of 1.88 (range: 0.46–2.94) for CHIKV in temperate areas from 9 different studies using different methodologies [32], which is consistent with our estimates.

The value of these estimates can be discussed with regard to different hypotheses, such as in particular the choice to consider the absence of asymptomatic infections. The proportion of CHIKV asymptomatic infections is usually estimated between 3 and 25% and remains highly variable across outbreaks [33]. A meta-analysis suggest that the proportion of inapparent CHIKV infections is lineage dependent and that less inapparent infections are associated with the ECSA lineage than the Asian lineage [34], which is the lineage incriminated in the two events reported here. The decision not to consider the occurrence of asymptomatic forms was mainly supported by the significant investigation effort deployed around the two transmission foci (door-to-door case search, awareness of health professionals from the area, and communication in the local media), which strongly reduces the probability of paucisymptomatic cases that would not have been identified. Moreover, a model allowing to integrate different values for the proportion of asymptomatic forms was also considered in order to evaluate the impact on the estimation of the parameters. This sensitivity analysis shows that the impact of taking into account a proportion of asymptomatic infections remains limited (S1 Text). Based on these different elements, the choice to consider only symptomatic cases was made with a view to meeting the decision-making issues discussed here, while keeping the model as simple as possible. However, this choice could be reconsidered for other objectives or even other epidemiological situations, such as large-scale epidemics.

Our study highlights the influence of factors such as the month of introduction of the virus and the delay of vector control measures. The comparison between the two events suggests that the number of cases expected in the absence of treatment is strongly influenced by the date of occurrence, which can effectively be considered as a proxy of vector population dynamics. The results based on standard dynamics allow to assess the relative importance of the various factors of interest (response time, date of start of the epidemic). The results of the model are also consistent with the two chikungunya outbreaks that occurred in Italy. They can partially explain the larger size of the Italian episodes that arose earlier in the season in Emilia-Romagna and Lazio [35,36] and for which the vector control measures were implemented approximately 2 months after the introduction of the primary case [35,37]. Under unfavourable conditions (peak vector activity and high vector densities), the potential for viral transmission remains high, even in the event of a rapid response, within 30 days. This observation justifies the need for increased surveillance during this period and the prompt implementation of preventive measures to reduce vector populations immediately after introduction of an infected case, to prevent autochthonous transmission. However, these results cannot be considered to have a 100% predictive value, since a climatic anomaly, such as heavy rainfall, is always possible and can have an impact on vector populations, as was observed in Montpellier in 2014 [26]. Our results on vector control sequences must be considered with caution, in particular those related to the spacing between consecutive treatments. Indeed, in this case, the dynamics of the populations of vectors depend strongly on the assumption we made regarding the recolonization of the treated areas. This fact underlines the need for a better understanding of recolonization after treatment. From an operational point of view, this dearth of knowledge justifies daily entomological monitoring of adult mosquito populations during all events of autochthonous transmission, not only to improve knowledge of these particular situations, but also to trigger vector control measures if the vector population increases.

Further developments in our model may be considered for the future. These include, in particular, the inclusion of climatic and environmental data for the real-time estimation of vector populations [38], and taking into account the age structure of the vector populations, another critical driver of transmission dynamics [39–41]. Scaling-up of the model is also important. Our model was developed based on two real-world transmission events which occurred in single patches of less than 10 ha, and assumed homogenous mixing within each patch. The definition of a spatially explicit metapopulation model would enable the spread of the virus at larger scales [42].

Quantitative results cannot be extrapolated to other viruses transmitted by *Ae*. *albopictus* (dengue, Zika). Human and vector susceptibilities to infections, extrinsic and intrinsic incubation periods, and the duration and level of viraemia are key parameters of virus transmission and may differ substantially from one virus to another. Recent events of dengue virus circulation suggest that this vector system is less efficient than that involving CHIKV [3]. However, we can reasonably assume similar conclusions in qualitative terms. Caution is likewise required in extending these conclusions to all CHIKV genotypes, as vector competence for a specific pathogen is governed by complex interactions between vector population, virus genotype and environmental conditions [43]. Experimental studies suggest a higher risk for ECSA strains to emerge in Europe, compared to Asian CHIKV strains [44]. These ECSA strains include CHIKV genotypes harbouring an alanine at position 226 of the E1 glycoprotein (i.e. without the E1–A226V mutation known for increasing virus replication and transmission by *Ae*. *albopictus*) as highlighted by the 2017 outbreak in Italy [45].

This modelling-based work emphasizes the importance of, and advocates for early detection of imported cases and timely biological confirmation of autochthonous cases to ensure timely vector control measures. In addition, given that vector density is a critical parameter for transmission after introduction, the preventive reduction of the size of vector populations is of outmost importance. To that end, awareness and mobilization of all involved stakeholders remain key elements in the control of vector-borne diseases: travellers, patients, doctors and laboratory workers for early detection and reporting, public health authorities and vector control agencies for the timely implementation of control measures, and the general public for routine control of breeding sites. Finally, our results also justify the current strategy of implementing vector control measures around imported cases, in a preventive manner, before any autochthonous transmission.

## Supporting information

S1 Text Parameter inference for Chikungunya virus outbreaks for different rates of asymptomatic infections. (DOCX)

S1 Table Transitions and rates of the stochastic model. (DOCX)

S2 Table Epidemiological and vector control original data of the outbreaks in Montpellier and Le-Cannet-des-Maures. (DOCX)

S3 Table State variables for the model used and initial conditions for chikungunya virus transmission events in Montpellier and Le-Cannet-des-Maures. (DOCX)

S1 Fig Parameter inference for Chikungunya virus outbreaks in Montpellier and Le Cannet-des-Maures. The different colours represent the traces of the 5 chains. Results for Montpellier are presented in the left-hand column whereas results for Le Cannet-des-Maures are shown in the right-hand column.
(TIF)

S2 Fig Stochastic simulations of the epidemic in the absence of vector control measures. Results for Montpellier are shown in purple and results for Le-Cannet-des-Maures are shown in blue.
(TIF)

S3 Fig Deterministic simulation of the evolution of the event in the absence of vector control measures. Results for Montpellier are shown in purple and results for Le-Cannet-des-Maures are shown in blue.
(TIF)

S4 Fig Simulations of the number of autochthonous cases expected as a function of the date of primary case introduction and delay of intervention for a standard mosquito population dynamic. Simulations were performed for two different vector control efficacy (Eff., in columns) and three different vector densities (in rows). For each setting, a sequence of 10 vector control treatments spaced 7 days apart is implemented.
(TIF)

S5 Fig Simulations of the number of autochthonous cases expected as a function of the date of primary case introduction and delay of intervention for a standard mosquito population dynamic. Simulations were performed for two different vector control efficacy (Eff., in columns) and three different vector densities (in rows). Vector controls spaced 7 days apart are performed provided that new cases occur.
(TIF)

S6 Fig Simulations of the mean number of autochthonous cases expected as a function of the date of primary case introduction for a standard mosquito population dynamic and different number of individual control measures. Simulations were performed for three different delays (in days) between successive vector control measures (dark grey labels), and four different response delays in vector control measures implementation (light grey labels). VCM: vector control measure(s).
(TIF)

## References

[1] Gould E, Pettersson J, Higgs S, Charrel R, de Lamballerie X. Emerging arboviruses: Why today? One Health. 2017;4: 1–13. doi: 10.1016/j.onehlt.2017.06.001 28785601 PMC5501887

[2] Jourdain F, Roiz D, de Valk H, Noël H, L’ambert G, Franke F, et al. From importation to autochthonous transmission: Drivers of chikungunya and dengue emergence in a temperate area. PLoS Negl Trop Dis. 2020;14: 1–19. doi: 10.1371/journal.pntd.0008320 32392224 PMC7266344

[3] Franke F, Giron S, Cochet A, Jeannin C, Leparc-Goffart I, de Valk H, et al. [Autochthonous chikungunya and dengue fever outbreak in Mainland France, 2010–2018]. Bull Epidémiol Hebd. 2019; 374–82.

[4] Bhatt S, Gething PW, Brady OJ, Messina JP, Farlow AW, Moyes CL, et al. The global distribution and burden of dengue. NATURE. 2013;496: 504–507. doi: 10.1038/nature12060 23563266 PMC3651993

[5] Amraoui F, Failloux AB. Chikungunya: an unexpected emergence in Europe. Curr Opin Virol. 2016;21: 146–150. doi: 10.1016/j.coviro.2016.09.014 27771517

[6] Lambrechts L, Scott TW, Gubler DJ. Consequences of the expanding global distribution of *Aedes albopictus* for dengue virus transmission. PLoS Negl Trop Dis. 2010;83: 6–7. doi: 10.1371/journal.pntd.0000646 20520794 PMC2876112

[7] Tsetsarkin KA, Chen R, Yun R, Rossi SL, Plante KS, Guerbois M, et al. Multi-peaked adaptive landscape for chikungunya virus evolution predicts continued fitness optimization in *Aedes albopictus* mosquitoes. Nat Commun. 2014;5: 4084. doi: 10.1038/ncomms5084 24933611 PMC7091890

[8] Schuffenecker I, Iteman I, Michault A, Murri S, Frangeul L, Vaney MC, et al. Genome microevolution of chikungunya viruses causing the Indian Ocean outbreak. PLoS Med. 2006;3: 1058–1070. doi: 10.1371/journal.pmed.0030263 16700631 PMC1463904

[9] Delisle E, Rousseau C, Broche B, Leparc-Goffart I, L’ambert G, Cochet A, et al. Chikungunya outbreak in Montpellier, France, september to october 2014. Eurosurveillance. 2015;20. doi: 10.2807/1560-7917.es2015.20.17.21108 25955774

[10] Calba C, Guerbois-Galla M, Franke F, Jeannin C, Auzet-Caillaud M, Grard G, et al. Preliminary report of an autochthonous chikungunya outbreak in France, July to September 2017. Eurosurveillance. 2017;22. doi: 10.2807/1560-7917.ES.2017.22.39.17-00647 29019313 PMC5709952

[11] Poletti P, Messeri G, Ajelli M, Vallorani R, Rizzo C, Merler S. Transmission potential of chikungunya virus and control measures: the case of Italy. PloS One. 2011;6: e18860. doi: 10.1371/journal.pone.0018860 21559329 PMC3086881

[12] Brady OJ, Johansson MA, Guerra CA, Bhatt S, Golding N, Pigott DM, et al. Modelling adult *Aedes aegypti* and *Aedes albopictus* survival at different temperatures in laboratory and field settings. Parasit Vectors. 2013;6. doi: 10.1186/1756-3305-6-351 24330720 PMC3867219

[13] Delatte H, Gimonneau G, Triboire A, Fontenille D. Influence of temperature on immature development, survival, longevity, fecundity, and gonotrophic cycles of *Aedes albopictus*, vector of chikungunya and dengue in the Indian Ocean. J Med Entomol. 2009;46: 33–41. doi: 10.1603/033.046.0105 19198515

[14] Vega-Rúa A, Zouache K, Girod R, Failloux A-BB, Lourenço-de-Oliveira R, Vega-Rua A, et al. High level of vector competence of *Aedes aegypti* and *Aedes albopictus* from ten American countries as a crucial factor in the spread of Chikungunya virus. J Virol. 2014;88: 6294–6306. doi: 10.1128/JVI.00370-14 24672026 PMC4093877

[15] Vazeille M, Moutailler S, Coudrier D, Rousseaux C, Khun H, Huerre M, et al. Two Chikungunya isolates from the outbreak of La Reunion (Indian Ocean) exhibit different patterns of infection in the mosquito, *Aedes albopictus*. PLoS One. 2007;2. doi: 10.1371/journal.pone.0001168 18000540 PMC2064959

[16] Christofferson RC, Chisenhall DM, Wearing HJ, Mores CN. Chikungunya viral fitness measures within the vector and subsequent transmission potential. PLoS One. 2014;9: e110538—e110538. doi: 10.1371/journal.pone.0110538 25310016 PMC4195746

[17] Rudolph KE, Lessler J, Moloney RM, Kmush B, Cummings DATT. Review article: Incubation periods of mosquito-borne viral infections: a systematic review. Am J Trop Med Hyg. 2014;90: 882–891. doi: 10.4269/ajtmh.13-0403 24639305 PMC4015582

[18] Thiberville S-DD, Moyen N, Dupuis-Maguiraga L, Nougairede A, Gould EA, Roques P, et al. Chikungunya fever: Epidemiology, clinical syndrome, pathogenesis and therapy. Antivir Res. 2013;99: 345–370. doi: 10.1016/j.antiviral.2013.06.009 23811281 PMC7114207

[19] Panning M, Grywna K, van Esbroeck M, Emmerich P, Drosten C. Chikungunya fever in travelers returning to Europe from the Indian Ocean region, 2006. Emerg Infect Dis. 2008;14: 416–22. doi: 10.3201/eid1403.070906 18325256 PMC2570846

[20] Burattini MN, Chen M, Chow A, Coutinho FAB, Goh KT, Lopez LF, et al. Modelling the control strategies against dengue in Singapore. Epidemiol Infect. 2008;136: 309–319. doi: 10.1017/S0950268807008667 17540051 PMC2870819

[21] Newton EA, Reiter P. A model of the transmission of dengue fever with an evaluation of the impact of ultra-low volume (ULV) insecticide applications on dengue epidemics. Am J Trop Med Hyg. 1992;47: 709–720. doi: 10.4269/ajtmh.1992.47.709 1361721

[22] Endo A, van Leeuwen E, Baguelin M. Introduction to particle Markov-chain Monte Carlo for disease dynamics modellers. Epidemics. 2019;29: 100363. doi: 10.1016/j.epidem.2019.100363 31587877

[23] R Core Development Team. R: A language and environment for statistical computing. R Foundation for Statistical Computing, Vienna, Austria. Version 3.5.3. 2019 [cited 13 Jun 2019]. https://www.r-project.org/

[24] Camacho A, Funk S. fitR: Tool box for fitting dynamic infectious disease models to time series. R package version 0.1. 2017.

[25] Lacour G, Chanaud L, L’Ambert G, Hance T. Seasonal Synchronization of Diapause Phases in *Aedes albopictus* (Diptera: Culicidae). Benoit JB, editor. PloS One. 2015;10: e0145311. doi: 10.1371/journal.pone.0145311 26683460 PMC4686165

[26] Roiz D, Boussès P, Simard F, Paupy C, Fontenille D. Autochthonous Chikungunya transmission and extreme climate events in Southern France. PLoS Negl Trop Dis. 2015;9: e0003854. doi: 10.1371/journal.pntd.0003854 26079620 PMC4469319

[27] Cianci D, Van Den Broek J, Caputo B, Marini F, Della Torre A, Heesterbeek H, et al. Estimating mosquito population size from mark-release-recapture data. J Med Entomol. 2013;50: 533–542. doi: 10.1603/me12126 23802447

[28] Feng X, Huo X, Tang B, Tang S, Wang K, Wu J. Modelling and Analyzing Virus Mutation Dynamics of Chikungunya Outbreaks. Sci Rep. 2019;9: 1–15. doi: 10.1038/s41598-018-37186-2 30814598 PMC6393467

[29] Dumont Y, Chiroleu F. Vector control for the chikungunya disease. Math Biosci Eng. 2010;7: 313–345. doi: 10.3934/mbe.2010.7.313 20462292

[30] Dumont Y, Chiroleu F, Domerg C. On a temporal model for the Chikungunya disease: Modeling, theory and numerics. Math Biosci. 2008;213: 80–91. doi: 10.1016/j.mbs.2008.02.008 18394655

[31] Manore CA, Hickmann KS, Xu S, Wearing HJ, Hyman JM. Comparing dengue and chikungunya emergence and endemic transmission in A. aegypti and A. albopictus. J Theor Biol. 2014;356: 174–191. doi: 10.1016/j.jtbi.2014.04.033 24801860 PMC4109365

[32] Liu Y, Lillepold K, Semenza JC, Tozan Y, Quam MBM, Rocklöv J. Reviewing estimates of the basic reproduction number for dengue, Zika and chikungunya across global climate zones. Environ Res. 2020;182: 109114. doi: 10.1016/j.envres.2020.109114 31927301

[33] Staples JE, Breiman RF, Powers AM. Chikungunya fever: an epidemiological review of a re-emerging infectious disease. Clin Infect Dis. 2009;49: 942–948. doi: 10.1086/605496 19663604

[34] Bustos Carrillo F, Collado D, Sanchez N, Ojeda S, Lopez Mercado B, Burger-Calderon R, et al. Epidemiological Evidence for Lineage-Specific Differences in the Risk of Inapparent Chikungunya Virus Infection. J Virol. 2018/11/23 ed. 2019;93. doi: 10.1128/JVI.01622-18 30463967 PMC6364014

[35] Rezza G, Nicoletti L, Angelini R, Romi R, Finarelli A, Panning M, et al. Infection with chikungunya virus in Italy: an outbreak in a temperate region. Lancet. 2007;370: 1840–1846. doi: 10.1016/S0140-6736(07)61779-6 18061059

[36] Riccardo F, Venturi G, Di Luca M, Del Manso M, Severini F, Andrianou X, et al. Secondary Autochthonous Outbreak of Chikungunya, Southern Italy, 2017. Emerg Infect Dis. 2019;25: 2093–2095. doi: 10.3201/eid2511.180949 31625839 PMC6810187

[37] Venturi G, Di Luca M, Fortuna C, Remoli ME, Riccardo F, Severini F, et al. Detection of a chikungunya outbreak in Central Italy, August to September 2017. Eurosurveillance. 2017;22. doi: 10.2807/1560-7917.ES.2017.22.39.17-00646 29019306 PMC5709953

[38] Tran A, L’Ambert G, Lacour G, Benoît R, Demarchi M, Cros M, et al. A rainfall- and temperature-driven abundance model for *Aedes albopictus* populations. Int J Env Res Public Health. 2013;10: 1698–1719. doi: 10.3390/ijerph10051698 23624579 PMC3709343

[39] Hardy JL, Houk EJ, Kramer LD, Reeves WC. Intrinsic factors affecting vector competence of mosquitoes for arboviruses. Annu Rev Entomol Vol 28. 1983;28: 229–262. doi: 10.1146/annurev.en.28.010183.001305 6131642

[40] Mayton EH, Tramonte AR, Wearing HJ, Christofferson RC. Age-structured vectorial capacity reveals timing, not magnitude of within-mosquito dynamics is critical for arbovirus fitness assessment. Parasit Vectors. 2020;13: 310. doi: 10.1186/s13071-020-04181-4 32539759 PMC7296759

[41] Armstrong PM, Ehrlich HY, Magalhaes T, Miller MR, Conway PJ, Bransfield A, et al. Successive blood meals enhance virus dissemination within mosquitoes and increase transmission potential. Nat Microbiol. 2020;5: 239–247. doi: 10.1038/s41564-019-0619-y 31819213 PMC7199921

[42] Moulay D, Pigne Y. A metapopulation model for chikungunya including populations mobility on a large-scale network. J Theor Biol. 2013;318: 129–139. doi: 10.1016/j.jtbi.2012.11.008 23154189

[43] Zouache K, Fontaine A, Vega-Rua A, Mousson L, Thiberge JM, Lourenco-De-Oliveira R, et al. Three-way interactions between mosquito population, viral strain and temperature underlying chikungunya virus transmission potential. Proc Biol Sci. 2014;281. doi: 10.1098/rspb.2014.1078 25122228 PMC4150320

[44] Vega-Rúa A, Marconcini M, Madec Y, Manni M, Carraretto D, Gomulski LM, et al. Vector competence of *Aedes albopictus* populations for chikungunya virus is shaped by their demographic history. Commun Biol. 2020;3. doi: 10.1038/s42003-020-1046-6 32581265 PMC7314749

[45] Lindh E, Argentini C, Remoli ME, Fortuna C, Faggioni G, Benedetti E, et al. The Italian 2017 outbreak chikungunya virus belongs to an emerging *Aedes albopictus*-adapted virus cluster introduced from the Indian subcontinent. Open Forum Infect Dis. 2019;6. doi: 10.1093/ofid/ofy321 30697571 PMC6345083

