# Supplementary material for: Estimating chikungunya virus transmission parameters and vector control effectiveness highlights key factors to mitigate arboviral disease outbreaks
Source: PLoS Negl Trop Dis. 2022 Mar 4;16(3):e0010244. doi: 10.1371/journal.pntd.0010244 (PMC8896662; doi:10.1371/journal.pntd.0010244)
Supplement: S1 Text — (DOCX) [file pntd.0010244.s001.docx]

**S1 Text**. Parameter inference for Chikungunya virus outbreaks for different rates of asymptomatic infections.

In this model, infected individuals ($E_{h}$) become, at a rate *ω_h_*, infectious and symptomatic ($I_{h}$) with the probability *p_s_*, or infectious and asymptomatic ($I_{h}^{a}$) with the probability (1 - *p_s_*). Finally, infectious individuals become recovered ($R_{h}$) at rate *σ*, whether they are symptomatic or asymptomatic (Figure a). All other transition rates and compartments are fully described in the main article.


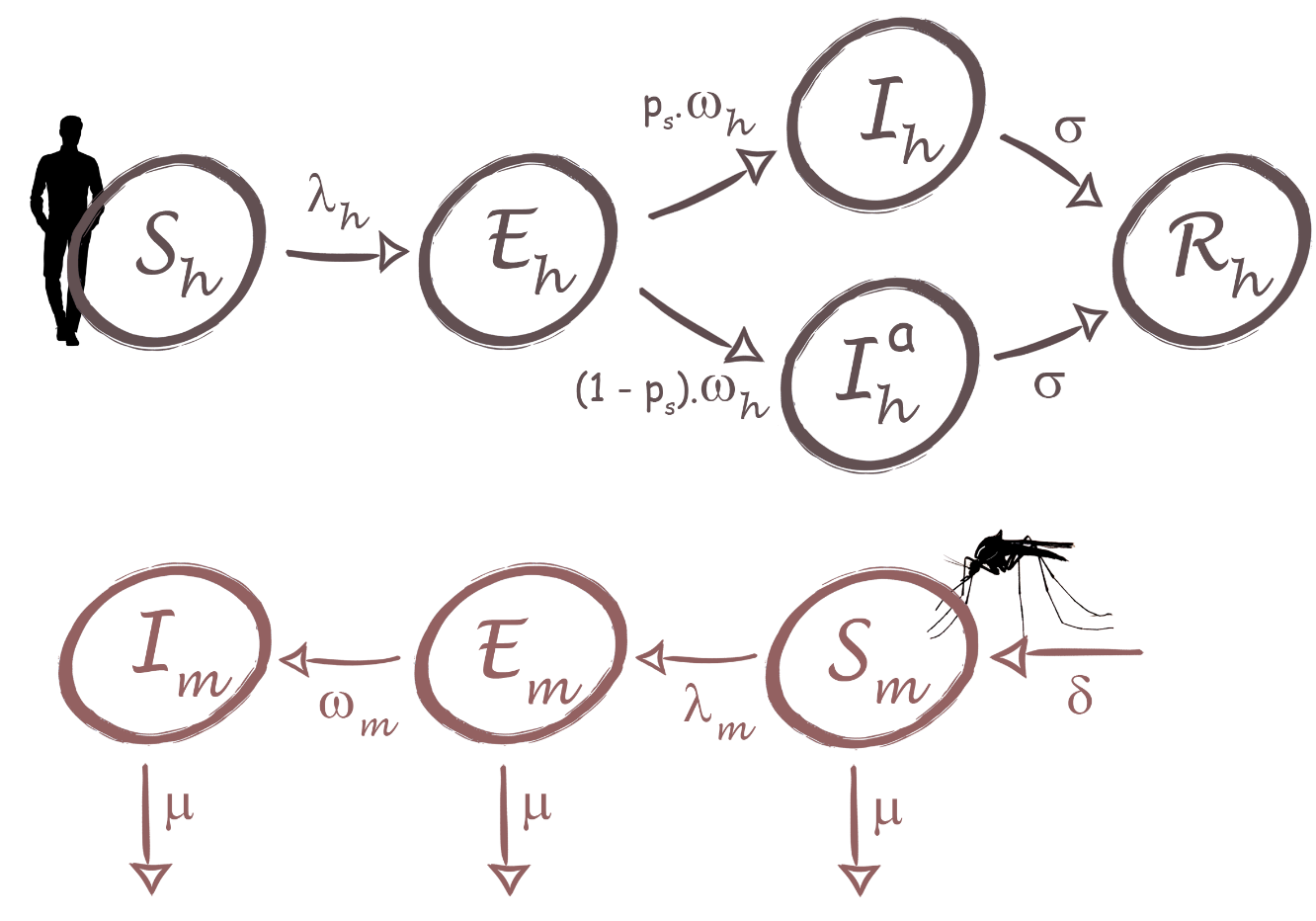


Figure a. Structure of the SEI-SEIR vector-host model considering asymptomatic hosts.

Considering, the rates of asymptomatic infections reported in the literature, two different values were selected for *p_s_*, the probability to be symptomatic..

**Table a. Estimates of the efficacy of vector control measures (Eff), the probability of host infection (b) and the corresponding basic reproduction rate (**$\mathcal{R}_{\mathbf{0}}$**) for both chikungunya events, according to different ratios of asymptomatic infections.**

| **Symptomatic infection ratio** | **Transmission event** | ***Eff*** | | |  | **Host infection probability (b)** | | |  | $\mathcal{R}_{\boldsymbol{0}}$ | | |
| --- | --- | --- | --- | --- | --- | --- | --- | --- | --- | --- | --- | --- |
|  |  | **Mean** | **5^th^ perc.** | **95^th^ perc.** |  | **Mean** | **5^th^ perc.** | **95^th^ perc.** |  | **Mean** | **5^th^ perc.** | **95^th^ perc.** |
| 100 % | Montpellier | 0.97 | 0.91 | > 0.99 |  | 0.34 | 0.33 | 0.35 |  | 1.86 | 1.83 | 1.88 |
|  | Le-Cannet-des-Maures | 0.83 | 0.78 | 0.89 |  | 0.29 | 0.28 | 0.31 |  | 1.78 | 1.72 | 1.84 |
| 90% | Montpellier | 0.97 | 0.91 | > 0.99 |  | 0.36 | 0.35 | 0.36 |  | 1.90 | 1.88 | 1.92 |
|  | Le-Cannet-des-Maures | 0.84 | 0.79 | 0.90 |  | 0.32 | 0.30 | 0.33 |  | 1.84 | 1.78 | 1.90 |
| 80% | Montpellier | 0.97 | 0.91 | > 0.99 |  | 0.37 | 0.36 | 0.38 |  | 1.94 | 1.92 | 1.96 |
|  | Le-Cannet-des-Maures | 0.85 | 0.80 | 0.91 |  | 0.33 | 0.31 | 0.35 |  | 1.89 | 1.84 | 1.95 |
