# Supplementary material for: Estimating chikungunya virus transmission parameters and vector control effectiveness highlights key factors to mitigate arboviral disease outbreaks
Source: PLoS Negl Trop Dis. 2022 Mar 4;16(3):e0010244. doi: 10.1371/journal.pntd.0010244 (PMC8896662; doi:10.1371/journal.pntd.0010244)
Supplement: S1 Table — (DOCX) [file pntd.0010244.s002.docx]

**S1 Table**. Transitions and rates of the stochastic model.

| **Transition** | **Rate** |
| --- | --- |
| S_h_ → S_h_ - 1 | $ab\frac{S_{h}I_{m}}{N_{h}}$ |
| E_h_ → E_h_ + 1 | $ab\frac{S_{h}I_{m}}{N_{h}}$ |
| E_h_ → E_h_ - 1 | $\omega_{h}E_{h}$ |
| I_h_ → I_h_ + 1 | $\omega_{h}E_{h}$ |
| I_h_ → I_h_ - 1 | $\sigma I_{h}$ |
| R_h_ → R_h_ + 1 | $\sigma I_{h}$ |
| S_m_ → S_m_ + 1 | $\delta N_{m}$ |
| S_m_ → S_m_ - 1 | $(ac\frac{I_{h}}{N_{h}}+\mu{)S}_{m}$ |
| E_m_ → E_m_ + 1 | $ac\frac{S_{m}I_{h}}{N_{h}}$ |
| E_m_ → E_m_ - 1 | $\left( \mu+\omega_{m} \right)E_{m}$ |
| I_m_ → I_m_ + 1 | $\omega_{m}E_{m}$ |
| I_m_ → I_m_ - 1 | $\mu I_{m}$ |
