# Supplementary material for: Estimating chikungunya virus transmission parameters and vector control effectiveness highlights key factors to mitigate arboviral disease outbreaks
Source: PLoS Negl Trop Dis. 2022 Mar 4;16(3):e0010244. doi: 10.1371/journal.pntd.0010244 (PMC8896662; doi:10.1371/journal.pntd.0010244)
Supplement: S2 Table — (DOCX) [file pntd.0010244.s003.docx]

### S2 Table. Epidemiological and vector control original data of the outbreaks in Montpellier and Le-Cannet-des-Maures.

| **Montpellier** | | |  |  | **Le-Cannet-des-Maures** | | |  |
| --- | --- | --- | --- | --- | --- | --- | --- | --- |
| **Date** | **t ^(1)^** | **Cases ^(2)^** | **VC** |  | **Date** | **t ^(1)^** | **Cases ^(2)^** | **VC** |
| 30/08/2014 | 0 | 0 |  |  | 10/07/2017 | 0 | 0 |  |
| 08/09/2014 | 9 | 1 |  |  | 28/07/2017 | 18 | 1 |  |
| 14/09/2014 | 15 | 3 |  |  | 02/08/2017 | 23 | 2 |  |
| 16/09/2014 | 17 | 4 |  |  | 03/08/2017 | 24 | 3 |  |
| 18/09/2014 | 19 | 5 |  |  | 07/08/2017 | 28 | 4 |  |
| 20/09/2014 | 21 | 7 |  |  | 11/08/2017 | 32 | 5 | 1 |
| 24/09/2014 | 25 | 8 |  |  | 14/08/2017 | 35 | 7 |  |
| 09/10/2014 | 40 | 9 |  |  | 17/08/2017 | 38 | 8 |  |
| 11/10/2014 | 42 | 10 |  |  | 18/08/2017 | 39 | 8 | 1 |
| 12/10/2014 | 43 | 11 |  |  | 19/08/2017 | 40 | 9 |  |
| 20/10/2014 | 51 | 11 | 1 |  | 22/08/2017 | 43 | 9 | 1 |
| 22/10/2014 | 53 | 12 |  |  | 25/08/2017 | 46 | 10 |  |
| 25/10/2014 | 56 | 12 | 1 |  | 29/08/2017 | 50 | 10 | 1 |
| 31/10/2014 | 62 | 12 | 1 |  | 30/08/2017 | 51 | 11 |  |

^(1)^ t=0 corresponds to the date of symptoms onset of the imported primary case. ^(2)^ Cumulated cases at date of symptom onset. VC: vector control, or day during which mosquito control measures were implemented.
