# Supplementary material for: Estimating chikungunya virus transmission parameters and vector control effectiveness highlights key factors to mitigate arboviral disease outbreaks
Source: PLoS Negl Trop Dis. 2022 Mar 4;16(3):e0010244. doi: 10.1371/journal.pntd.0010244 (PMC8896662; doi:10.1371/journal.pntd.0010244)
Supplement: S3 Table — (DOCX) [file pntd.0010244.s004.docx]

### S3 Table. State variables for the model used and initial conditions for chikungunya virus transmission events in Montpellier (2014) and Le-Cannet-des-Maures (2017).

| State | Montpellier | Le Cannet-des-Maures |
| --- | --- | --- |
| Sm | 4260 | 3444 |
| Em | 0 | 0 |
| Im | 1 | 1 |
| Sh | 497 | 378 |
| Eh | 0 | 0 |
| Ih | 0 | 0 |
| Rh | 0 | 0 |
| Size (ha) | 7.1 | 8.4 |
