# Supplementary figures and images for: Estimating chikungunya virus transmission parameters and vector control effectiveness highlights key factors to mitigate arboviral disease outbreaks
Source: PLoS Negl Trop Dis. 2022 Mar 4;16(3):e0010244. doi: 10.1371/journal.pntd.0010244 (PMC8896662; doi:10.1371/journal.pntd.0010244)

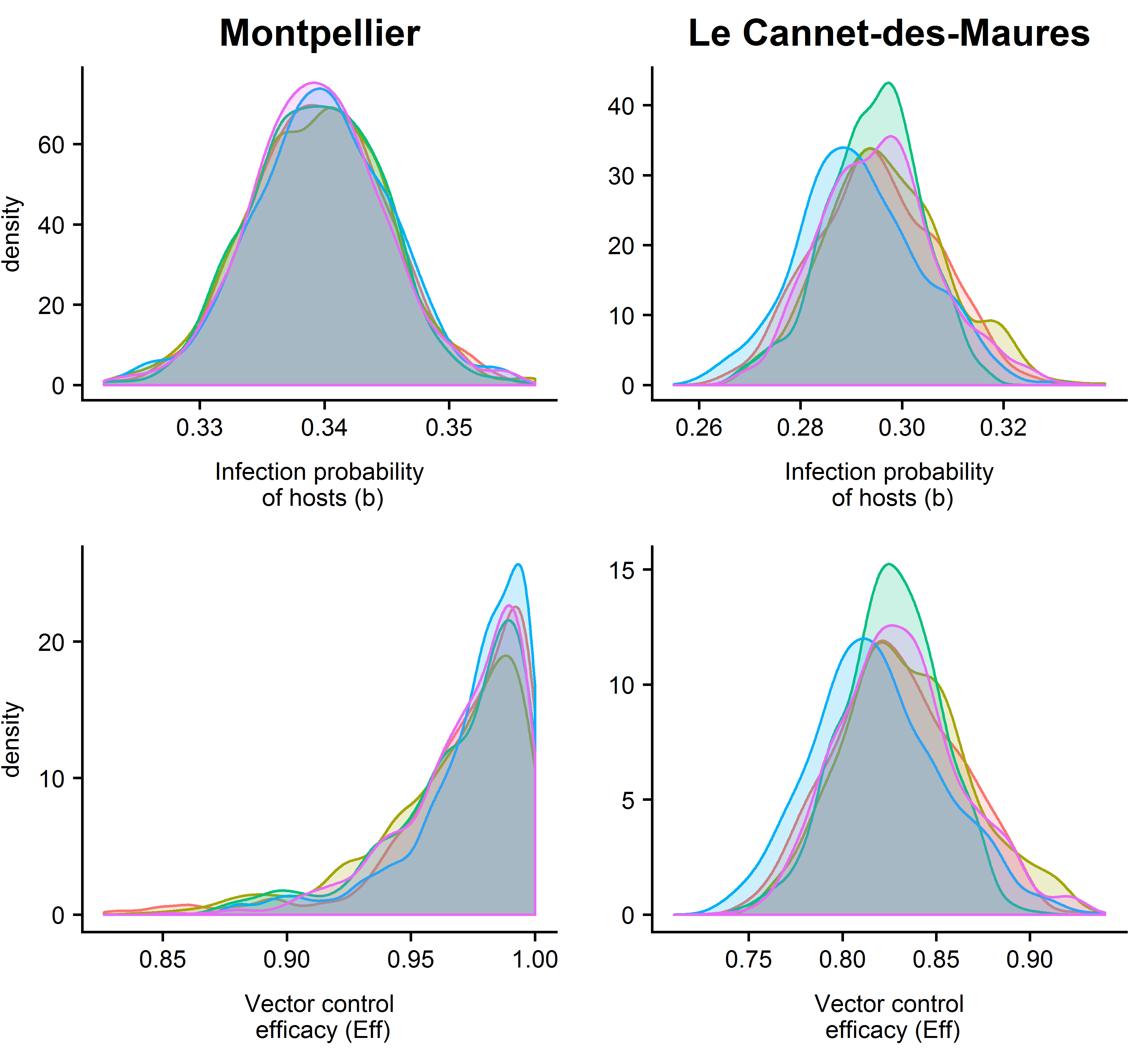

Supplement: S1 Fig — The different colours represent the traces of the 5 chains. Results for Montpellier are presented in the left-hand column whereas results for Le Cannet-des-Maures are shown in the right-hand column. (TIF) [file pntd.0010244.s005.tif]

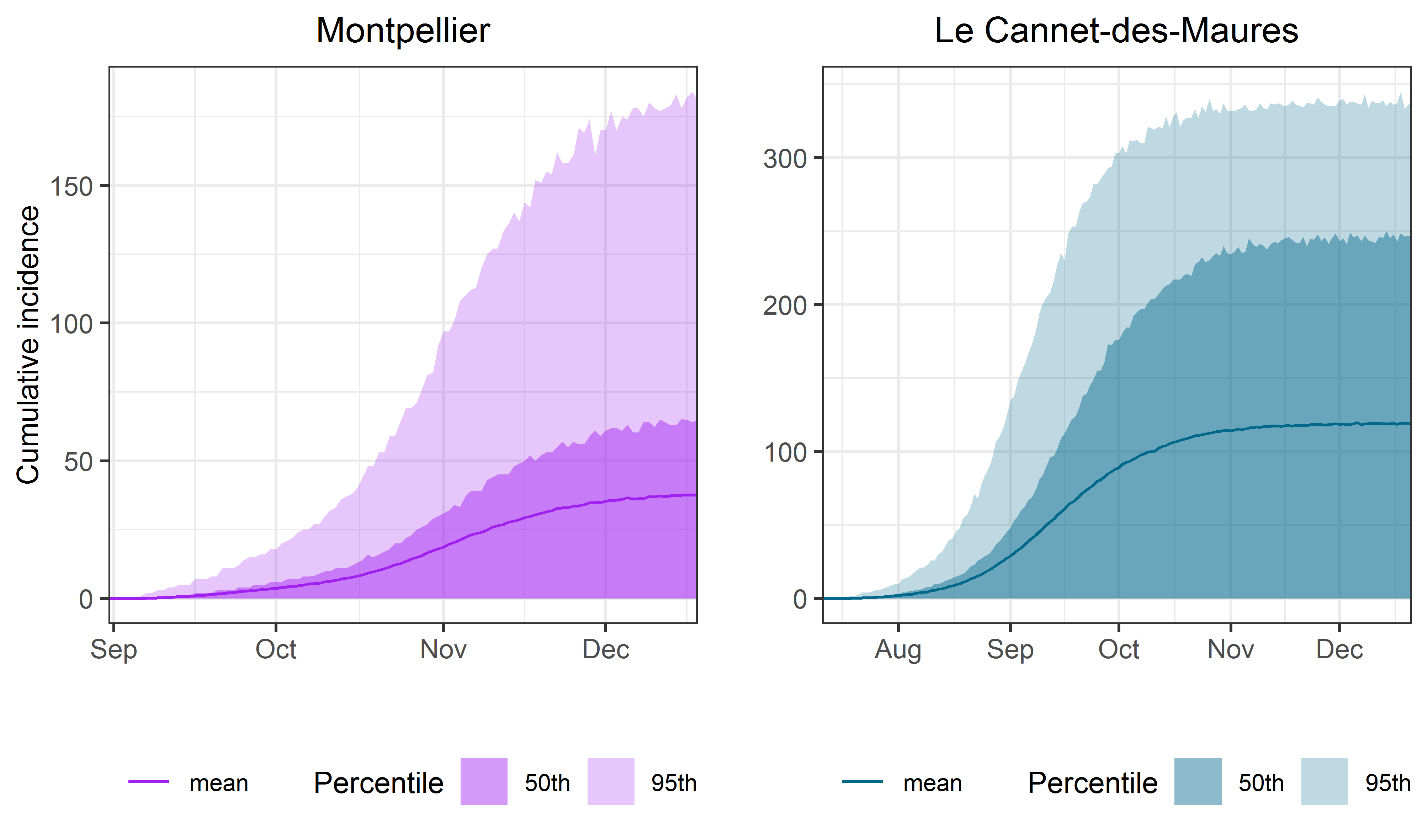

Supplement: S2 Fig — Results for Montpellier are shown in purple and results for Le-Cannet-des-Maures are shown in blue. (TIF) [file pntd.0010244.s006.tif]

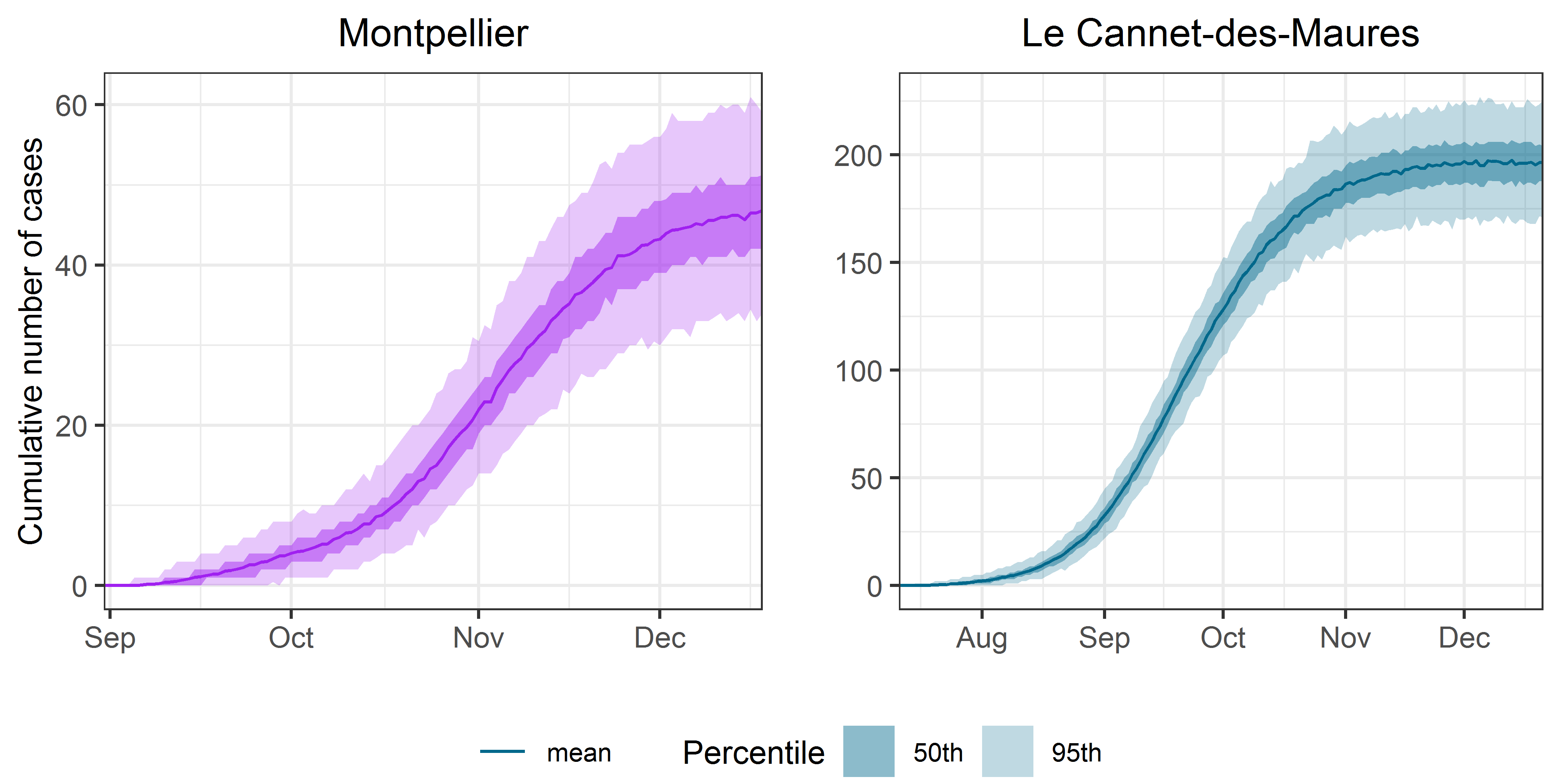

Supplement: S3 Fig — Results for Montpellier are shown in purple and results for Le-Cannet-des-Maures are shown in blue. (TIF) [file pntd.0010244.s007.tif]

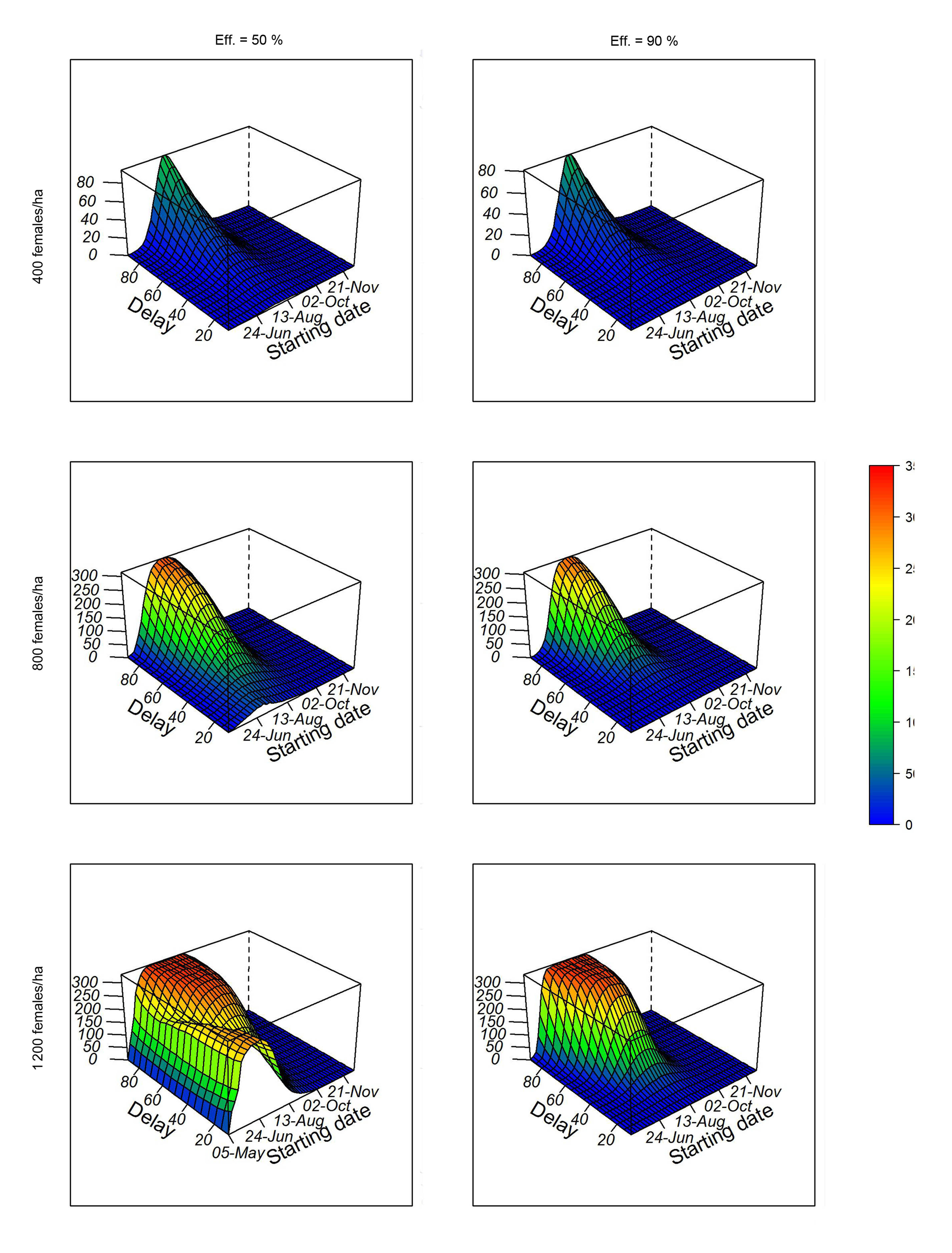

Supplement: S4 Fig — Simulations were performed for two different vector control efficacy (Eff., in columns) and three different vector densities (in rows). For each setting, a sequence of 10 vector control treatments spaced 7 days apart is implemented. (TIF) [file pntd.0010244.s008.tif]

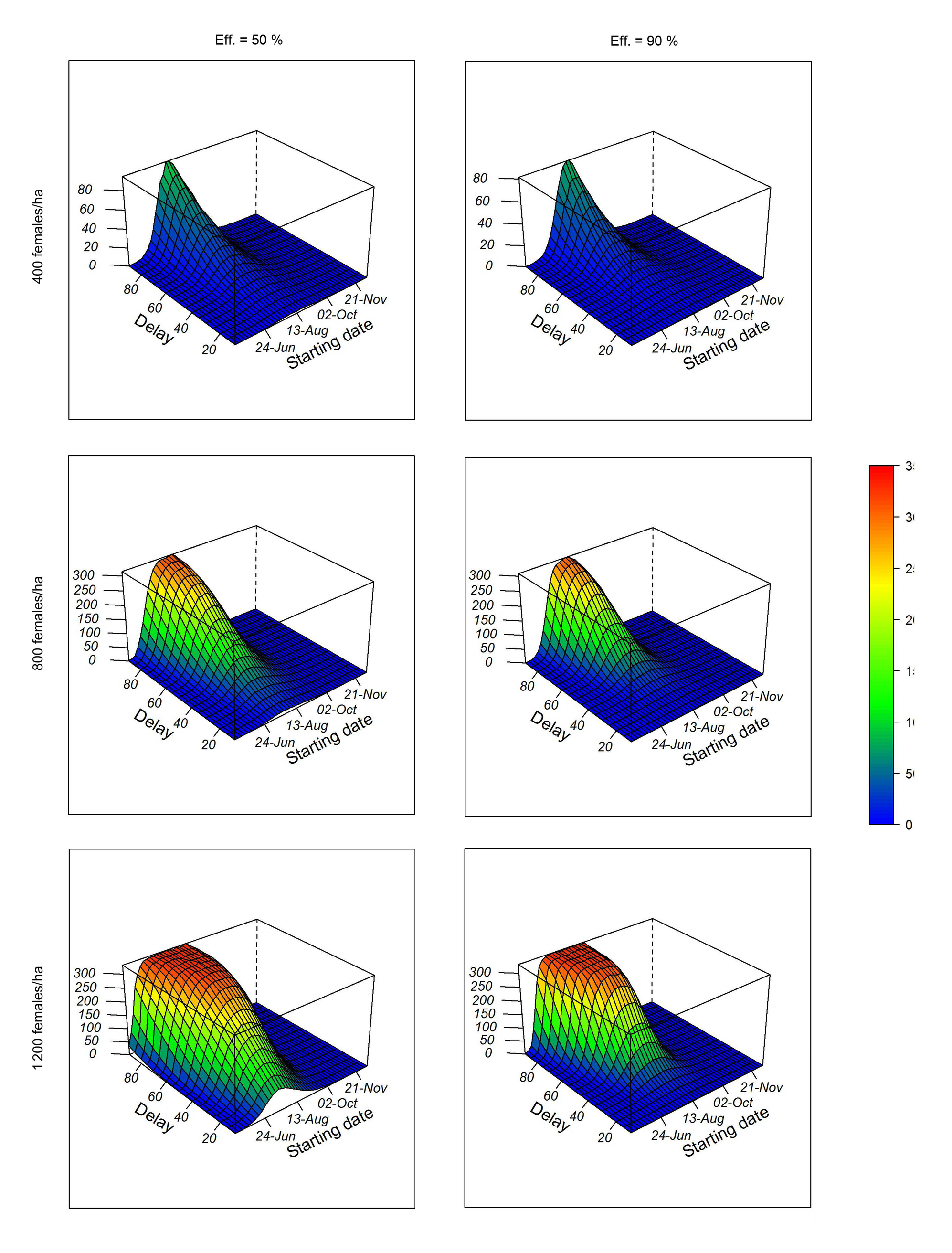

Supplement: S5 Fig — Simulations were performed for two different vector control efficacy (Eff., in columns) and three different vector densities (in rows). Vector controls spaced 7 days apart are performed provided that new cases occur. (TIF) [file pntd.0010244.s009.tif]

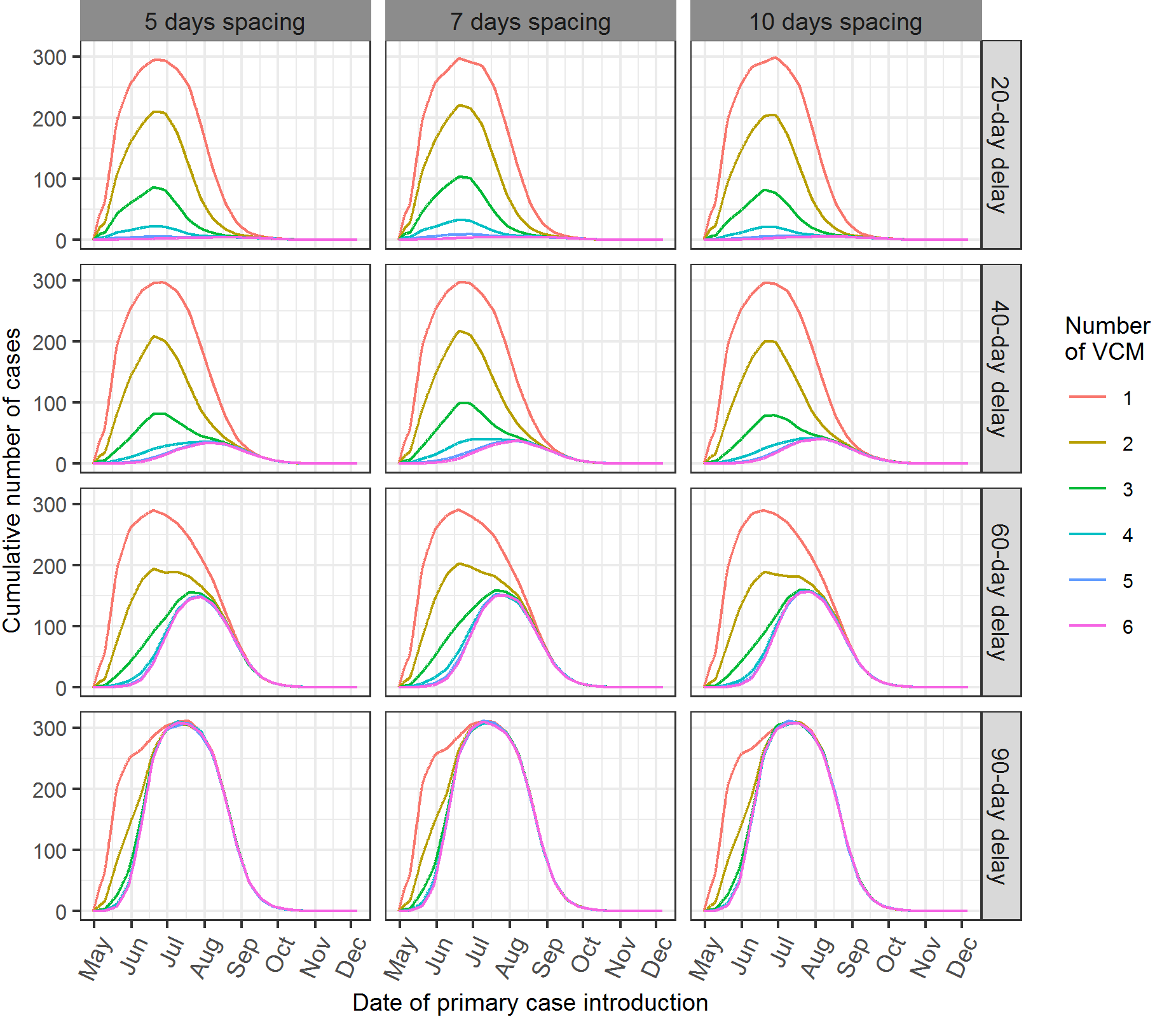

Supplement: S6 Fig — Simulations were performed for three different delays (in days) between successive vector control measures (dark grey labels), and four different response delays in vector control measures implementation (light grey labels). VCM: vector control measure(s). (TIF) [file pntd.0010244.s010.tif]
